# Supplementary material for: Causal interplay between lactose intolerance and gut microbiota: a combined bidirectional Mendelian randomization and in vivo validation study
Source: Front Nutr. 2026 Jun 1;13:1803337. doi: 10.3389/fnut.2026.1803337 (PMC13265576; doi:10.3389/fnut.2026.1803337)
Supplement: Supplementary file 1 [file Data_Sheet_1.zip › supplementary materials/Forward/forest plot/ebi-a-GCST90027717.finngen_R12_E4_LACTONAS.pdf]

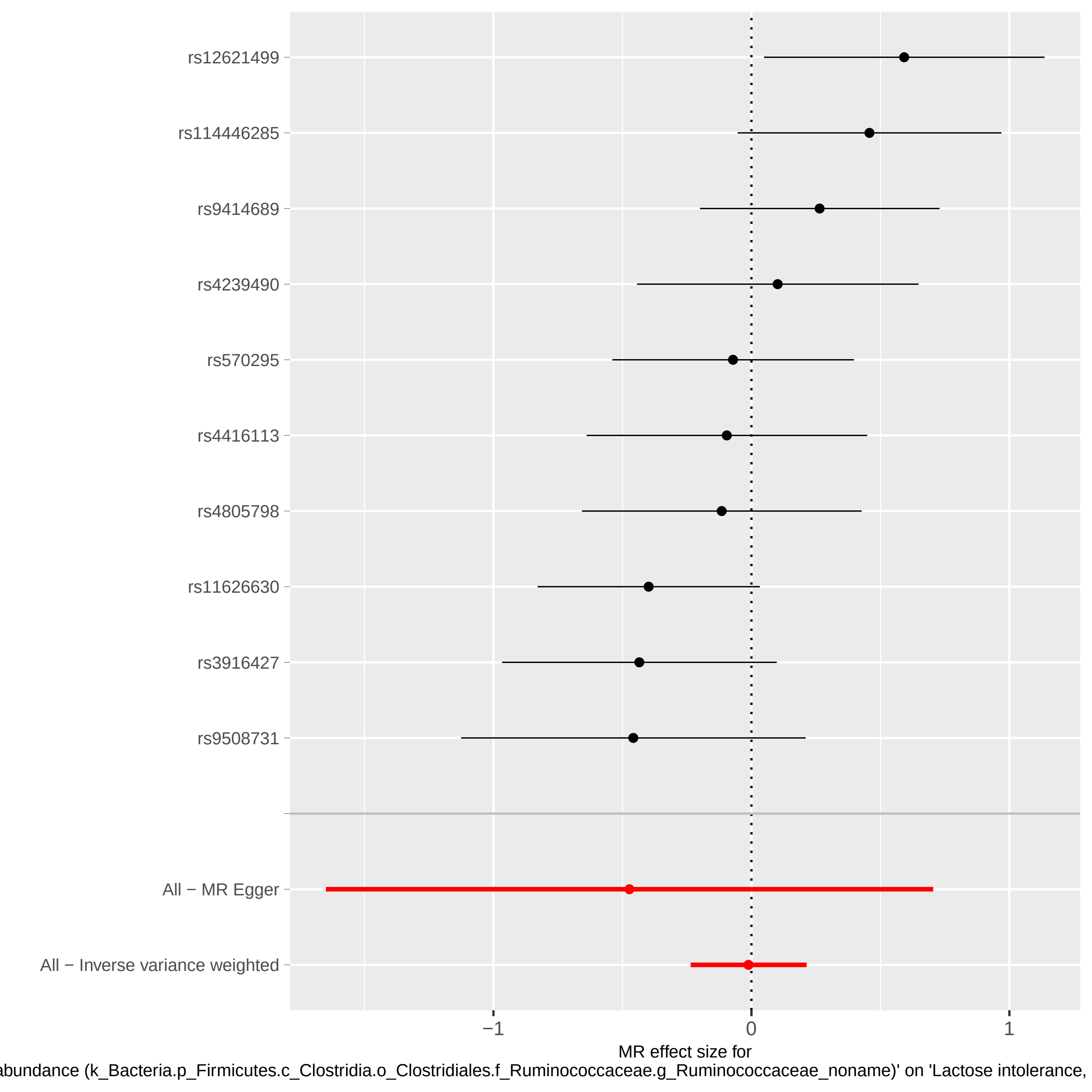

rs12621499
rs114446285
rs9414689
rs4239490
rs570295
rs4416113
rs4805798
rs11626630
rs3916427
rs9508731
All − MR Egger
All − Inverse variance weighted
−1
0
1
MR effect size for
abundance (k_Bacteria.p_Firmicutes.c_Clostridia.o_Clostridiales.f_Ruminococcaceae.g_Ruminococcaceae_noname)' on 'Lactose intolerance,
